# Protocol for automated production of human stem cell derived liver spheres

**DOI:** 10.1016/j.xpro.2021.100502

**Published:** 2021-04-30

**Authors:** Jose Meseguer-Ripolles, Alvile Kasarinaite, Baltasar Lucendo-Villarin, David C. Hay

**Affiliations:** 1 Centre for Regenerative Medicine, Institute for Regeneration and Repair, The University of Edinburgh, Edinburgh BioQuarter, 5 Little France Drive, Edinburgh EH16 4UU, UK

**Keywords:** Metabolism, Stem Cells, Cell Differentiation, Tissue Engineering

## Abstract

This protocol describes how to produce human liver spheres from pluripotent stem cell-derived hepatic progenitors, endothelial cells, and hepatic stellate cells. Liver spheres form by self-assembly in microwells, generating up to 73 spheres per well of a 96-well plate. This process was automated using liquid handling and pipetting systems, permitting cost-effective scale-up and reducing sphere variability. In its current form, this system provides a powerful tool to generate human liver tissue for disease modeling and drug screening.

For complete details on the use and execution of this protocol, please refer to Lucendo-Villarin et al. (2020) (https://doi.org/10.1088/1758-5090/abbdb2).

## Highlights

• Protocol to produce and self-assemble hepatocytes, endothelial, and stellate cells
• Generation of an automated system for 3D liver sphere production
• Scalable production of phenotypically stable liver spheres for disease modeling

## Graphical abstract

**Figure:**
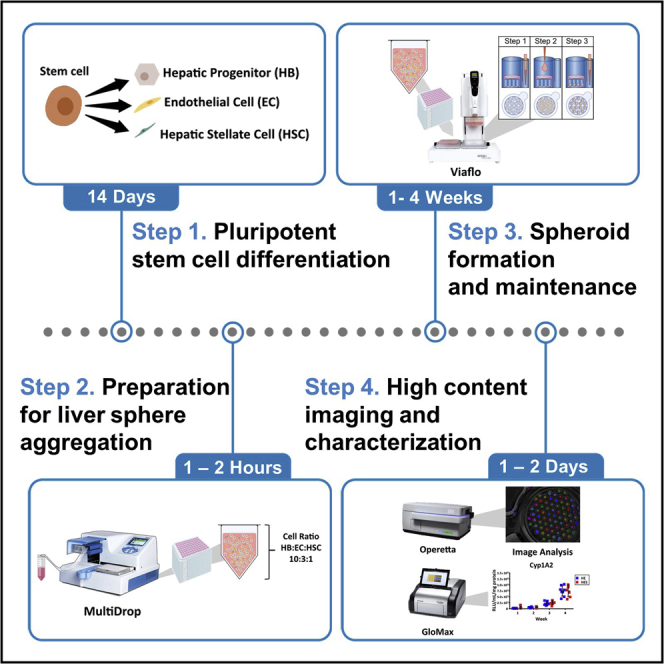

## Teaser

This protocol describes how to produce human liver spheres from pluripotent stem cell-derived hepatic progenitors, endothelial cells, and hepatic stellate cells. Liver spheres form by self-assembly in microwells, generating up to 73 spheres per well of a 96-well plate. This process was automated using liquid handling and automatic pipetting systems, permitting cost-effective scale-up and reducing sphere variability. In its current form, this system provides a powerful tool to generate human liver tissue for disease modeling and drug screening.

## Before you begin

### Planning stem cell differentiation timings

**Timing: 14 days**

To date, most *in vitro* models used to study liver biology are two-dimensional (2D) in nature. Stem cell based models offer a powerful tool for disease modeling or drug screening (Cayo et al., 2017; Lucendo-Villarin et al., 2017; Sinton et al., 2021). However, 2D systems do not fully recapitulate liver structure as well as interaction of multiple cell types. To address this, we have generated stem cell-derived liver spheroids containing key cell types found in the liver. This protocol allows the combination of three different stem cell-derived somatic cells (hepatic progenitor cells, endothelial cells, and hepatic stellate cells). Due to the difference in time to produce each cell type, the differentiation procedures need to be coordinated. Stellate cell differentiation commences first followed by hepatic progenitor differentiation then endothelial differentiation (Table 1). On the aggregation day, single cell collection of the three cell types is required to form the aggregates at specific cell ratios (Table 2).

***Note:*** For 1 x 96 well-plate of livers spheroids 4 x Petri dishes of hepatic progenitor cells, 4 wells of 6 well-plate of endothelial cells and 1 well of 6 well-plate of hepatic stellate cells are needed. Due to endothelial differentiation efficiency variability, it is recommended to prepare 2–4 extra wells to ensure the desired number of endothelial cells is obtained.

**Table 1:** Schematic representation of the differentiation timeline

Cell type	Differentiation timing (days)													Aggregation day
HSC	−1	0	1	2	3	4	5	6	7	8	9	10	11	12
HB				**−1**	**0**	**1**	**2**	**3**	**4**	**5**	**6**	**7**	**8**	**9**
EC								**−1**	**0**	**1**	**2**	**3**	**4**	**5**

HSC: Hepatic stellate cells, HB: Hepatic progenitors and EC: Endothelial cells.

The differentiation is achieved for the three different stem cell-derived cell types in parallel for liver sphere aggregation. Day -1 represents the day of the single cell seeding.

**Table 2:** Parameters required for the cell aggregation for a Gri3D® 96-well plate

Cell type	Cell density required (cells/mL)	Cells per micromold	Total cells per well	Total cells needed per plate	Volume (μL) needed per well to prepare 60 μL
HB	10950000	3000	219000	21024000	30
ELC	6570000	900	65700	6307200	15
HSC	2190000	300	21900	2102400	15

### Preparation of Laminin-521-coated plates

**Timing: 30 min**

1. Thaw a 100 μg/mL stock vial of recombinant laminin 521 (LN-521) at 4°C overnight (10 – 12 h).
2. Dilute the thawed LN-521 in ice-cold 1**×** DPBS (with Ca2+/Mg2+) to make a 5 μg/mL solution.
3. For a well of 6 well-plate, add 1 mL of the 5 μg/mL solution per well. For a petri dish of 55 cm^2^, add 5 mL of the 5 μg/mL solution. Ensure that the LN-521 solution is covering the whole surface.
4. Following LN-521 coating, incubate the plates or petri dishes in a 37°C/5% CO_2_ cell culture incubator for 2 h or at 4°C overnight (10–12 h) on a flat surface.
5. Store coated plates at 4°C on a flat surface. Seal the plates to reduce evaporation and use within two weeks.
6. Before use, allow the plate to warm up by placing the plate/petri dish in a 37°C/5% CO_2_ cell culture incubator for 30 min or warm up at room temperature (15°C–21°C) for 2 h.

**CRITICAL:** Do not let the LN-521 evaporate. Seal the plates with parafilm to avoid evaporation and to reduce the risk of contamination. Addition of an extra 1 mL of 1**×** DPBS (with Ca2+/Mg2+) can reduce the risk of evaporation. The use of LN-521 is recommended to obtain reproducible results. For other matrices, further optimisation in cell seeding densities will be required.

### Differentiation media and growth factor preparation

**Timing: 3 h**

***Note:*** Unless otherwise specified, all procedures should be performed using sterile techniques and working in a cell culture hood class II. All reconstituted medias should be used withing a month following preparation. If needed, media can be frozen −20°C and stored for 6 months, do not freeze again after thaw. Growth factors can be stored at −20°C for short term storage up to 6 months or at −80°C for long term storage for at least 12 months. Once thawed, growth factors should be used within two weeks. See key resources table section for growth factor preparations and Lucendo-Villarin et al., 2020, https://doi.org/10.1088/1758-5090/abbdb2

**CRITICAL:** Maintenance and the scaling-up of a human pluripotent stem cells (hPSC) stock population accordingly to meet the three differentiations is key to ensure sufficient cell numbers.

7. Stem cell media
  a. Add 100 mL mTeSR1™ 5**×** supplement to the 400 mL mTeSR1™ basal medium.
8. Differentiation media
  a. For hepatic cell differentiation:
    i. Prepare endoderm differentiation medium by adding 1% penicillin streptomycin and 1**×** B27 supplement to 500 mL RPMI 1640 medium.
    ii. Prepare hepatic progenitor differentiation medium by mixing 400 mL knockout (KO)-DMEM, 100 mL KOSR Serum Replacement, 1% non-essential amino acids, 1% DMSO, 1% penicillin streptomycin, 0.5% Glutamax and 0.2% β-mercaptoethanol.
    iii. Prepare hepatocyte maturation medium by combining 500 mL Hepato-ZYME, 10 μM hydrocortisone 21-hemisuccinate sodium salt (HCC), 1% penicillin streptomycin and 1% Glutamax.
  b. For endothelial cell differentiation:
    i. Prepare mesoderm priming medium N2B27 by mixing 250 mL DMEM:F12 with 250 mL CTS Neurobasal media supplemented with 2.5 mL GlutaMAX (100**×**), 10 mL CTS B27, 5 mL CTS N2, and 0.5 mL β-mercaptoethanol.
    ii. Reconstitute StemPro-34 SFM medium.
  c. For hepatic stellate cell differentiation:
    i. Prepare hepatic stellate cell (HSC) differentiation medium by combining 57% DMEM low glucose, 40% MCDB-201-water, 0.25× linoleic acid-bovine serum albumin, 0.25× insulin-transferrin-selenium, 1% penicillin streptomycin, 10−4 M L-ascorbic acid, 2.5 μM dexamethasone and 50 μM β -mercaptoethanol.
  d. For liver sphere culture:
    i. Prepare liver sphere medium by mixing 1:1 of William's E media and SFM-Endothelial media with 5% Serum replacement, 1% Glutamax and 1% penicillin-streptomycin.

## Key resources table

**Table:** 

REAGENT or RESOURCE	SOURCE	IDENTIFIER
**Antibodies**		

PE Mouse anti-Human CD144 (1:50 dilution)	BD Biosciences	560410
APC mouse anti-human CD31 (1:50 dilution)	eBioscience	17-0319-42
CD144 (VE-Cadherin) MicroBeads, human	Miltenyi Biotec	Cat#130-097-857

**Chemicals, peptides, and recombinant proteins**		

B27	Life Technologies	Cat#12587-010
B27 CTS	Life Technologies	Cat#17504044
β-Mercaptoethanol	Life Technologies	Cat#31350-010
Bovine serum albumin	Sigma-Aldrich	Cat#A2058
CHIR-99021	Sigma-Aldrich	Cat#SML-1046-5MG
CTS Neurobasal media	Life Technologies	Cat#A13712-01
Dexamethasone	Sigma-Aldrich	Cat#D1756-25MG
Dimethyl sulfoxide, DMSO	Life Technologies	Cat#D5879
DMEM low glucose	Thermo Fisher	Cat#11885084
DMEM/F-12, GlutaMAX	Life Technologies	Cat#31331-028
DPBS with Calcium and Magnesium	Thermo Fisher	Cat#14040133
DPBS, no calcium, no magnesium	Thermo Fisher	Cat#14190250
Forskolin	Sigma-Aldrich	Cat#F-6886-10MG
Gentle Cell Dissociation Reagent	STEMCELL Technologies	Cat#7174
GlutaMAX-I	Life Technologies	Cat#35050-038
Hepatocyte growth factor	PeproTech	Cat#100-39
HepatoZYME-SFM	Life Technologies	Cat#17705-021
Human basic fibroblast growth factor	PeproTech	Cat#100-18B
Human Endothelial SFM	Thermo Fisher	Cat#11111044
Human epithelial growth factor	PeproTech	Cat#AF-100-15-100UG
Human recombinant BMP4	R&D	Cat#314-BP
Human recombinant FGF-1	PeproTech	Cat#100-17A
Human recombinant FGF-3	PeproTech	Cat#160-05
Human recombinant laminin 521	BioLamina	Cat#LN521-02
Human recombinant VEGF protein	R&D	Cat#293-VE
Hydrocortisone 21-hemisuccinate sodium salt	Sigma-Aldrich	Cat#H4881-1G
Insulin-transferrin-sodium selenite media supplement	Sigma-Aldrich	Cat#I1884-1VL
Knockout DMEM	Life Technologies	Cat#10829-018
KO-SR (Knockout serum replacement)	Life Technologies	Cat#10828-028
L-Ascorbic acid	Sigma-Aldrich	Cat#A4544-25G
Linoleic acid	Sigma-Aldrich	Cat#L9655-5ML
MCDB-201-water	Sigma-Aldrich	Cat#M6770-1L
Minimal essential medium non-essential amino acids	Life Technologies	Cat#11140-035
mTeSR1 Medium	STEMCELL Technologies	Cat#85850
N2 CTS	Life Technologies	Cat#A13707-01
Oncostatin M	PeproTech	Cat#300-10
Palmitic acid	Sigma-Aldrich	Cat#P0500-10G
Penicillin-streptomycin (10,000 U/mL)	Life Technologies	Cat#15140-122
Recombinant Human/Mouse/Rat Activin A Protein	R&D	Cat#338-AC
Retinol	Sigma-Aldrich	Cat#R7632-100MG
Rho-associated kinase (ROCK) inhibitor Y27632	Tocris Bioscience	Cat#1254
RPMI 1640	Life Technologies	Cat#11875-093
StemPro-34	Life Technologies	Cat#10639-011
TrypLE Express Enzyme (1**×**), no phenol red	Life Technologies	Cat#12604-021
William's E Medium, no phenol red	Thermo Fisher	Cat#A1217601
Wnt3a	R&D	Cat#1324-WN/CF

**Critical commercial assays**		

BCA Protein Assay Kit	Thermo Fisher	Cat#23225
ELISA Kit ALBUMIN	Alpha Diagnostics	Cat#1190
ELISA Kit AFP	Alpha Diagnostics	Cat#500
P450-Glo CYP1A2 Assay and Screening System	Promega	Cat#V8771

**Experimental models: cell lines**		

Human embryonic stem cell line H9	WiCell	WA09
Human embryonic stem cell line Man12	University of Manchester	N/A
Human iPSC line p106	WiCell	JHU106i

**Software and algorithms**		

Columbus software	PerkinElmer	https://www.perkinelmer.com/uk/product/image-data-storage-and-analysis-system-columbus

**Other**		

96-Well plate Gri3D	SUN bioscience	Cat#Gri3D-96
VWR® 96-Well Deep Well Plates	VWR	Cat# 10755-248
GloMax explorer multiplex plate reader	Promega	Cat#GM3500
GripTips for VIAFLO 96	Integra	Cat#6434
LS Columns	Miltenyi Biotec	Cat#130-042-401
MACS MultiStand	Miltenyi Biotec	Cat#130-042-303
MultidropCombi Reagent Dispenser	Thermo Fisher	Cat#5840300
XUBA 1 ultrasonic bath	Xuba	Cat# XUBA1
Operetta High-Content Imaging System	PerkinElmer	Cat#HH12000000
Small Cell Scraper, Non-pyrogenic, Sterile	Corning Incorporated Costar	Cat#3010
Standard tube dispensing cassette	Thermo Fisher	Cat#24072670
VIAFLO 96 Electronic 96-channel pipette	Integra	Cat#6001
White plates for CYP assays	Greiner Bio-One	Cat#655075

## Materials and equipment

**Table:** 

CELL MEDIA PREPARATION – Storage at 4°C for a month		
Reagent	Final concentration	Amount needed (for 500 mL)
**Stem cell medium**		

mTeSR1™ 5**×** supplement	1**×**	100 mL
mTeSR1™ basal medium	N/A	400 mL


**Hepatic differentiation media**		

**Endoderm differentiation medium**		
Penicillin streptomycin (100**×**)	1%	5 mL
B27 supplement (50**×**)	1%	10 mL
RPMI 1640 medium	N/A	500 mL
**Hepatic progenitor differentiation medium**		
β-mercaptoethanol (50 mM)	0.2%	1 mL
GlutaMAX-I (100**×**)	0.5%	2.5 mL
DMSO (100**×**)	1%	5 mL
Penicillin streptomycin (100**×**)	1%	5 mL
Minimal essential medium Non-Essential Amino Acids	1%	5 mL
KOSR serum replacement	20%	100 mL
Knockout (KO)-DMEM	N/A	400 mL
**Hepatocyte maturation medium**		
GlutaMAX-I (100**×**)	1%	5 mL
Penicillin streptomycin (100**×**)	1%	5 mL
Hydrocortisone 21-hemisuccinate sodium salt (HCC; 1 mM stock)	10 μM	5 mL
Hepato-ZYME	N/A	500 mL

**Endothelial differentiation media**		

**Mesoderm priming medium N2B27**		
β-mercaptoethanol (50 mM)	0.1%	0.5 mL
GlutaMAX-I (100**×**)	0.5%	2.5 mL
CTS N2 (100**×**)	1%	5 mL
CTS B27 (2%)	0.04%	10 mL
DMEM:F12, GlutaMAX-I	50%	250 mL
CTS Neurobasal media	50%	250 mL
**Endothelial cell differentiation medium SP34**		
Penicillin streptomycin (100**×**)	1%	5 mL
StemPro-34	N/A	500 mL

**Hepatic stellate cell differentiation medium**		

β-mercaptoethanol (50 mM)	50 μM	0.5 mL
Dexamethasone (2.5 mM stock)	2.5 μM	0.5 mL
L-ascorbic acid (100 mM stock)	10^-4^ M	0.5 mL
Insulin-transferrin-sodium selenite	0.25**×**	1.25 mL
Linoleic acid-bovine serum albumin	0.25**×**	1.25 mL
Penicillin streptomycin (100**×**)	1%	5 mL
MCDB-201-water	40%	200 mL
DMEM Low Glucose	57%	285 mL
**Liver sphere culture**		
GlutaMAX-I	1%	5 mL
Penicillin streptomycin (100**×**)	1%	5 mL
KOSR serum replacement	10%	55 mL
Human Endothelial-SFM	50%	250 mL
William’s E medium, no phenol red	50%	250 mL

**Table:** 

Growth factor and other reagents preparation					
Reagent	Solute	Solvent	Stock concentration	Working concentration	Storage
Human activin A	Human activin A lyophilized protein	Sterile 0.2% bovine serum albumin (BSA)/DPBS	100 μg/mL	100 ng/mL	−80°C in small aliquots; 6 months
Wnt3A	Mouse Wnt3A lyophilized protein	Sterile 0.2% BSA/DPBS	10 μg/mL	50 ng/mL	−80°C in small aliquots; 6 months
Hydrocortisone 21-hemisuccinate sodium salt (HCC)	HCC	PBS	10 μM	100 nM	Filter the solution with sterile 0.22 μm filters; −20°C in 5 mL aliquots. 6 months
Human hepatocyte growth factor (HGF)	Human HGF lyophilized protein	Sterile 0.2% BSA/DPBS	10 μg/mL	10 ng/mL	−80°C in small aliquots, 12 months
Epithelial growth factor (EGF)	Human EGF lyophilized protein	Sterile 0.2% BSA/DPBS	10 μg/mL	10 ng/mL	−80°C in small aliquots, 12 months
Basic fibroblast growth factor (bFGF)	Human bFGF lyophilized protein	Sterile 0.2% BSA/DPBS	10 μg/mL	10 ng/mL	−80°C in small aliquots, 6 months
Vascular endothelial growth factor (VEGF)	Human VEGF lyophilized protein	Sterile 0.2% BSA/DPBS	10 μg/mL	50 ng/mL (except with SP34 media use at 200 ng/mL)	−80°C in small aliquots; 6 months
Oncostatin M (OSM)	OSM	Sterile 0.2% BSA/DPBS	20 μg/mL	20 ng/mL	−80°C in small aliquots, 12 months
Y-27632 (ROCKi)	Rho-associated Kinase (ROCK) Inhibitor Y27632	Sterile 0.2% BSA/DPBS	10 mM	10 μM	−80°C in small aliquots; 1 month
CHIR-99021	CHIR-99021	Sterile DMSO	10 μM	0.7 μM	−80°C in small aliquots; 12 months
BMP4	Human recombinant BMP4	Sterile 0.2% BSA/DPBS	50 μg/mL	25 ng/mL	−80°C in small aliquots; 6 months
Forskolin	Forskolin	Sterile 0.2% BSA/DPBS	10 mM	2 μM	−80°C in small aliquots; 6 months
FGF-1	Human recombinant FGF-1	Sterile 0.2% BSA/DPBS	20 μg/mL	20 ng/mL	−80°C in small aliquots; 6 months
FGF-3	Human recombinant FGF-3	Sterile 0.2% BSA/DPBS	20 μg/mL	20 ng/mL	−80°C in small aliquots; 6 months
Retinol	Retinol	Sterile 0.2% BSA/DPBS	500 mM	Dilute 1:100 in EtOH and use at 5 μM	−80°C in small aliquots. 6 months
Palmitic Acid	Palmitic acid	Sterile EtOH	100 mM	100 μM	−20°C in small aliquots, 6 months

## Step-by-step method details

### Passaging human pluripotent stem cells (hPSCs) for differentiation

**Timing: 2 h**

Day -1

This step describes how to prepare hPSCs from colony to single-cell suspension which will be used for differentiation into other cell types. This protocol requires a starting population of hPSCs colonies in a 6 well plate format to be in 75%–85% confluency. A single cell solution is required to prepare the hPSCs for differentiation into the three cell types required for the liver spheroid aggregation. The protocol was adapted from (Wang et al., 2017), (Meseguer-Ripolles et al., 2018) and (Wang et al., 2019).

1. Aspirate the medium from hPSCs at 75%–85% confluency
2. Wash the cells with 1 mL of 1**×** room temperature room temperature (15°C–21°C) DPBS (without Ca2+/Mg2+) per well of a 6 well-plate
3. Aspirate the DPBS from the well
4. Add 1 mL of Gentle Cell Dissociation Reagent per well and leave the cells at in 37°C/5% CO_2_ cell culture incubator for 5–8 min to dissociate into single cells

***Note:*** Incubation time can vary depending on cell confluency or cell line used

5. Prepare the new plates by carefully aspirating the 5 μg/mL solution in DPBS of a pre-warmed 6 well-plate without damaging the laminin-coated surface
6. Immediately add 1 mL of stem cell medium consisting of pre-warmed supplemented mTeSR1™ with 10 μM Rho-associated kinase (ROCK) inhibitor Y-27632 per well of a 6 well-plate (half a volume of media)

***Note:*** The use of ROCKi Y-27632 enhances cell attachment and survival.

7. Place the LN-521 coated plates with medium back to in 37°C/5% CO_2_ cell culture incubator until the cells are ready

**CRITICAL:** Do not allow LN-521 coated wells to dry.

8. To check if the cells have dissociated into single cells, observe via microscope, and increase the incubation time if necessary

***Note:*** Do not incubate the gentle cell dissociation reagent for more than 15 min

9. Aspirate the gentle cell dissociation reagent and immediately add 1 mL of pre-warmed supplemented mTeSR1™ with 10 μM ROCKi Y-27632 per well of a 6 well-plate to stop the reaction
10. De-attach the cells into the medium from the well using a cell scraper
11. Collect the cell medium with the cells into a 15 mL centrifuge tube
12. Centrifuge the cells at 0.2 x g for 5 min at room temperature
13. Aspirate the supernatant carefully without disturbing the cell pellet

***Note:*** Tap the cell pellet to ensure that there are no cell clumps.

14. Add 5-10 mL of pre-warmed supplemented mTeSR1™ with 10 μM ROCKi Y-27632 to the cells and gently pipette up and down to ensure a homogeneous single cell solution
15. Count the cells using an automatic cell counter using trypan blue to exclude death cells
16. Seed the cells by adding the volume needed for the specific cell density described on Table 2 into the LN-521 coated well with mTeSR1™ with 10 μM ROCKi Y-27632 and top up with supplemented mTeSR1™ with 10 μM ROCKi Y-27632 to a final volume of 2 mL per well in a 6 well plate

***Note:*** this procedure will be used to prepare stem cell derived hepatocytes, endothelial, and stellate cells at different timepoints. Cell densities required for each cell type are different and they are described on Table 2 and Lucendo-Villarin et al., 2020, https://doi.org/10.1088/1758-5090/abbdb2

**CRITICAL:** To ensure an even distribution of the cell suspension, move gently the plate ten times back and forth and left to right.

17. Incubate the plate in a 37°C/5% CO_2_ cell culture incubator for 24 h to start the differentiation to either hepatocytes, endothelial or stellate cells

### Hepatic stellate cell differentiation

**Timing: [14 days, 1h daily]**

This step describes how to differentiate hPSCs into hepatic stellate cells. This protocol generates homogeneous hepatic stellate cells. This protocol was adapted from (Coll et al., 2018).

18. Aspirate the 5 μg/mL solution of LN-521 in DPBS from a pre-warmed (37°C – 15 min) 6 well-plate.
19. Add 1 mL of mTeSR1 ™ supplemented with 10 μM Y-27632 per well of a 6 well-plate.
20. Prepare a single-cell suspension of 75,000 cells/cm^2^ as described before and add 1 mL per well. Dispense the cell suspension evenly in the well.
21. Incubate over night at 37°C/5% CO_2_

**CRITICAL:** To ensure an even distribution of the cell suspension, shake gently the plate ten times back and forth and left to right.

22. After 24 h, replace the medium with 2 mL per well of HSC differentiation medium supplemented with 20 ng/mL of BMP4. Incubate at 37°C/5% CO_2_. Replenish the medium every 48 h for four days.
23. On day 4, change the medium with HSC differentiation medium supplemented with 20 ng/mL of FGF1 and 20 ng/mL of FGF3.
24. On day 6, change the medium with HSC differentiation medium supplemented with 20 ng/mL of FGF1, 20 ng/mL of FGF3, 5 μM retinol and 100 μM palmitic acid.
25. On day 8, change the medium with HSC differentiation medium supplemented with 5 μM retinol and 100 μM palmitic acid. Replenish the media every 48 h until day 12.
26. To collect the cells for sphere aggregation, wash once with DPBS 1**×** and dispense 1 mL of prewarmed TrypLE. Incubate for 3–6 min at 37 °C/5% CO_2_ to obtain a single cell suspension.
27. Once cells detach, collect the cell suspension into a 50 mL tube, wash the wells with 5 mL of cold liver sphere medium supplemented with 10 μM Y-27632 to collect any leftover cells and transfer the media into the tube containing the cell suspension. Ensure a single cell suspension by filtering the cell suspension through a 30 μm filter.
28. Count the cells using an automatic cell counter using trypan blue to exclude death cells.

***Note:*** Manual cell count is not recommended as it can introduce user-to-user variability.

29. Spin down cell suspension by centrifuging the cells at 0.2 x g for 10 min.
30. Resuspend the cell pellet at 2.19 x 10^6^ cells/mL in liver sphere medium supplemented with 10 μM Y-27632, 10 ng/mL EGF, 10 ng/mL FGF, 10 ng/mL HGF, 20 ng/mL OSM and 50 ng/mL VEGF.

***Note:*** A second count after cell resuspension is recommended to ensure the right cell concentration is achieved.

31. Keep the cell suspension at 4°C up to 4 h until all cell types are ready for the liver sphere aggregation step.

***Note:*** Cells can be fixed for FACS characterization at this stage.

### Hepatic progenitor cell differentiation

**Timing: [11 days, 1h daily]**

This step describes how to differentiate hPSCs into hepatic cells. This protocol generates homogeneous hepatic cells. This protocol was previously described in (Wang et al., 2017).

32. Aspirate the 5 μg/mL solution of LN-521 in DPBS from a pre-warmed (37°C–15 min) 6 well-plate.
33. Add 1 mL of mTeSR1 ™ supplemented with 10 μM Y-27632 per well of a 6 well-plate.
34. Prepare a single-cell suspension of 40,000 cells/cm^2^ as described before and add 1 mL per well. Dispense the liquid evenly in the well when pipetting.

**CRITICAL:** To ensure an even distribution of the cell suspension, shake gently the plate ten times back and forth and left to right. SEE ABOVE

35. After 24 h when cell confluency reaches 40%, replace the media with 2 mL per well of endoderm differentiation medium supplemented with 100 ng/mL of Activin A and 50 ng/mL Wnt3a. Incubate at 37°C/5% CO_2_. Replenish medium every 24 h for three days.
36. On day 3, change the medium with hepatic progenitor differentiation medium. Incubate at 37°C / 5% CO_2_. Replenish medium every 48 h for five days.
37. On day 8, change the medium with hepatocyte maturation medium supplemented with 10 ng/mL of HGF and 20 ng/mL of OSM. Incubate at 37°C/5% CO_2_.
38. To collect the cells for sphere aggregation on day 9, wash once with DPBS 1**×** and dispense 1 mL of prewarmed TrypLE. Incubate for 6–10 min at 37°C/5% CO_2_ to obtain a single cell suspension.
39. Once cells detach, collect the cell suspension into a 50 mL tube, wash the wells with 7 mL of cold liver sphere medium supplemented with 10 μM Y-27632 to collect any leftover cells and transfer the media into the tube containing the cell suspension. Ensure a single cell suspension by filtering the cell suspension through a 30 μm filter.
40. Count the cells using an automatic cell counter using trypan blue to exclude death cells.

***Note:*** Manual cell count is not recommended as it can introduce user-to-user variability.

41. Spin down cell suspension by centrifuging the cells at 0.2 x g for 10 min.
42. Resuspend the cell pellet at 10.95 x 10^6^ cells/mL in liver sphere medium supplemented with 10 μM Y-27632, 10 ng/mL EGF, 10 ng/mL FGF, 10 ng/mL HGF, 20 ng/mL OSM and 50 ng/mL VEGF.

***Note:*** A second count after cell resuspension is recommended to ensure the right cell concentration is achieved.

43. Keep the cell suspension at 4°C up to 4 h until all cell types are ready for the liver sphere aggregation step.

***Note:*** Cells can be fixed for FACS characterization at this stage.

### Endothelial cell differentiation

**Timing: 7 days, 1 h daily**

This step describes how to differentiate hPSC into endothelial cells. This protocol generates a non-homogeneous endothelial cell population ranging from 41% to 64% purity. To ensure a pure > 90% population of CD144/CD31 positive endothelial cells, magnetic cell separation (MACS) enrichment for CD144 is required. This protocol was adapted from (MacAskill et al., 2018)

44. Aspirate the 5 μg/mL solution of LN-521 in DPBS of a pre-warmed 6 well-plate.
45. Add 1 mL of mTeSR1 ™ supplemented with 10 μM Y-27632 per well of a 6 well-plate.
46. Prepare a single-cell suspension of 25,000 cells/cm^2^ as described before and add 1 mL per well. Dispense the liquid evenly in the well when pipetting.

**CRITICAL:** To ensure an even distribution of the cell suspension, shake gently the plate ten times back and forth and left to right.

47. After 24 h, replace the media with 3 mL per well of mesoderm priming medium supplemented with 10 μM CHIR99021 and 25 ng/mL of BMP4. Incubate for 72 h at 37°C/5% CO_2_.
48. Following the 72 h, change the medium to StemPro-34 media supplemented with 200 ng/mL of VEGF and forskolin 2 μM. Refresh the medium after 24 h.
49. To collect the cells for CD144 enrichment using MACS, wash X1 with DPBS 1**×** and dispense 1 mL of prewarmed TrypLE. Incubate for 3–6 min at 37°C/5% CO_2_ to obtain a single cell suspension.
50. Collect the cell suspension into a 50 mL tube, wash the wells with 5 mL of cold DPBS with 0.5% BSA to collect any leftover cells and transfer the media into the tube containing the cell suspension. Ensure a single cell suspension by filtering the cell suspension through a 30 μm filter.
51. Count the cells using an automatic cell counter using trypan blue to exclude death cells.

***Note:*** Counting is necessary to ensure that the CD144-magnetic bead labelling is performed according to manufacturer’s instructions. Manual cell count is not recommended as it can introduce user-to-user variability.

52. Spin down cell suspension by centrifuging the cells at 0.2 x g for 5 min.

***Note:*** Cells can be collected for FACS characterization of the impurified population for the endothelial markers CD31 and CD144, see materials and equipment table for details.

53. MACS enrichment is performed as recommended by manufacturers’ instructions. Briefly, resuspend cell pellet in 80 μL per 10^7^ cells and add 20 μL of CD144-magnetic beads. Mix well and incubate at 4°C for 15 min. Wash cells with 1–2 mL of cold DPBS with 0.5% BSA and centrifuge the cell suspension at 0.2 x g for 5 min.
54. Resuspend up to 10^8^ cells in 500 μL of DPBS with 0.5% BSA.
55. Place the MACS column in the magnetic field and prepare the column by rinsing it with 3 mL of DPBS with 0.5% BSA.
56. Apply the cell suspension onto the column, flow-through contains the un-labeled cells. Wash the column three times with 3 mL of DPBS with 0.5% BSA.

***Note:*** Cells can be collected for FACS characterization of the negative population.

57. Add 5 mL of DPBS with 0.5% BSA and quickly collect the magnetically labeled cells by pushing the plunger into the column.
58. Count the cells to calculate the yield of CD144 positive endothelial cells and spin them at 0.2 x g for 10 min.
59. Resuspend the cell pellet at 6.57 x 10^6^ cells/mL in liver sphere medium supplemented with 10 μM Y-27632, 10 ng/mL EGF, 10 ng/mL FGF, 10 ng/mL HGF, 20 ng/mL OSM and 50 ng/mL VEGF.

***Note:*** A second count after cell resuspension is recommended to ensure the right cell concentration is achieved.

60. Keep the cell suspension at 4°C up to 4 h until all cell types are ready for the liver sphere aggregation step.

***Note:*** Cells can be collected for FACS characterization of the positive population.

### 96-Well plate Gri3D® preparation

**Timing: 15 min**

**Figure 1:**
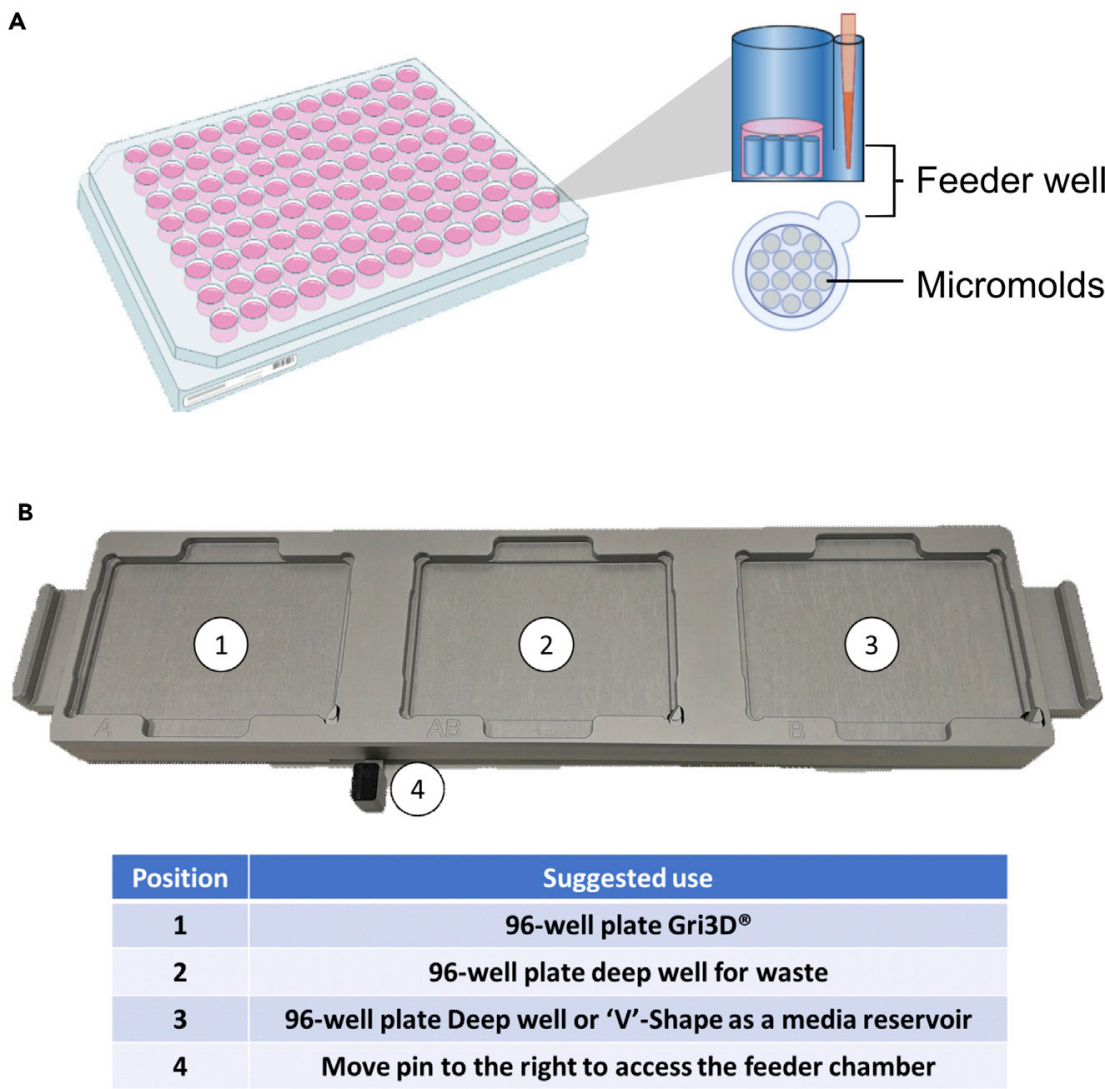
Schematic representation of the sphere production pipeline (A) Schematic representation of a well of a Gri3D_ plate. (B) Suggested use of the Viaflo plate station. Position 1 – for the 96-well plate Gri3D_ with the microwells (feeder wells) facing left. Position 2 - for a 96- deep well plate for the waste collection. Position 3 – for the ‘V’-shape plate with the cell suspension. Position 4 – for accessing the feeder well by moving the pin to the left.

This step describes how to prepare the 96-well plate Gri3D® for cell seeding. Each well of a 96-well plate Gri3D contains a Polyethylene glycol (PEG)- hydrogel with 73 micromolds of 500 μM of diameter. Upon adding a single-cell suspension in the wells, cells will be deposited into the micromolds by gravity where they will start to self-assemble into spheroids. Each well of the Gri3D plates, contains a feeder cavity or well which can be used for media change to reduce spheroid disruption (Figure 1A). Wells need to be washed prior spheroid formation to remove shipping buffer. By using automatic liquid handling systems, semi-automation of this process can be achieved. For this protocol we use both Multidop (Thermofisher) and Viaflo (Integra) systems. First, the multidrop is a high-speed dispenser compatible with 6- to 1536-well plates, it works by dispensing specific volumes into the wells in a column-based manner using dispensing cassettes which can vary depending on the volume to dispense, in our protocol we use standard dispensing cassettes. The Viaflo is a handheld 24, 96 or 384 channel programmable pipette that allows an increase throughput when working with multi-well plates. The Viaflo can be used with either a two- or three-plate station. The usage of the three-plate station minimizes the risk of contamination by reducing the need of changing plates when working under sterile conditions.

61. Place the 96-well plate Gri3D® into one of the plate holders of a three station Viaflo with a 96 well head. Place the plate with the A1 well facing the top left, the microwells or feeder wells should be facing left at this position (Figure 1A). Place a 96-deep well plate into the next position of the station and use it as a waste.
62. Prepare a 96-deep well plate with 900 μL of DPBS per well for Gri3D® plate to be prepared. This will serve as a reservoir of DPBS to wash three times the Gri3D® plates to prepare them for cell culture. Place the deep well plate with DPBS into the last Viaflo station available.

***Note:*** The use of a multidrop with a standard cassette to dispense the DPBS into the deep well can reduce time and variability.

63. Position the Viaflo head in the Gri3D® plate position, move the pin of the plate holder to the left to work in a 384-well plate mode. This mode should align the tips with the microwell of the 96-well plate Gri3D®.
64. To remove all the shipment buffer from the wells, aspirate 300 μL from the feeder well. Move the Viaflo head into the central position (Figure 1B) where the waste deep-well plate for waste is placed. Pin back to center to work in a 96-well plate mode and dispense the liquid into the waste plate.
65. Move the plate station to the right for the Viaflo to access the plate with DPBS. Aspirate 250 μL. Move the plate station to the right and move the pin to the left to work in a 384-well plate mode. Place the tips into the feeder well and dispense the 250 μL into the feeder well. Mix up and down 100 μL five times to ensure any reminder of the buffer from the Gri3D® plate is mixed with the DPBS.
66. This process is considered a wash, repeat it two more times to ensure full removal of the Gri3D® plate.

***Note:*** The volume DPBS dispensed into the deep-well should cover the three washes.

67. After the last wash, leave the DPBS into the plate and place it into the incubator at 37°C/5% CO_2_ to warm up the hydrogel from Gri3D® plate until the cell suspension for liver sphere aggregation is prepared.

### Automated liver sphere aggregation using 96-well plate Gri3D® plates

**Timing: 4 h**

**CRITICAL:** The three different cell lines need to be differentiated in parallel to achieve the last day of the differentiation to be ready to process on the day of the liver sphere aggregation (see also Table 1).

**Figure 2:**
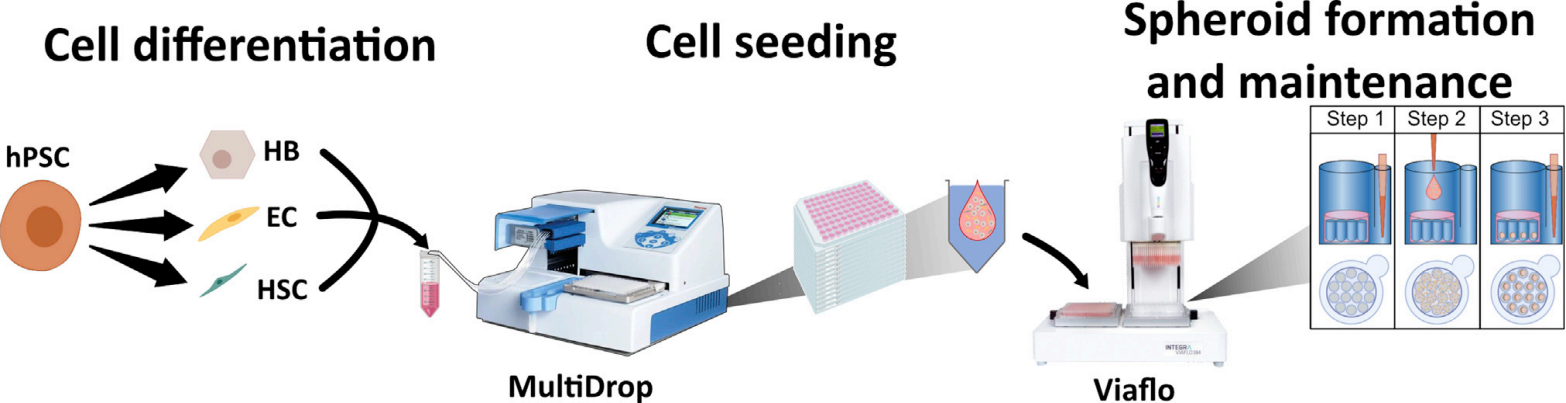
Schematic representation of the semi-automation pipeline to generate liver spheres After differentiation from hPSCs to hepatic progenitors, endothelial cells, and hepatic stellate cells, the cells were mixed at the specific ration (10:3:1 accordingly). The cell suspension containing three cell types was dispensed into a 96-well V shape plate using the multidrop automatic liquid handling system. The cells were seeded into the microwells using the Viaflo automatic pipette that was also used for spheroid maintenance. Adapted from B Lucendo-Villarin et al., 2020 Biofabrication https://doi.org/10.1088/1758-5090/abbdb2.

This step describes how to prepare the cell suspension with the three cell types for the liver sphere aggregation using the 96-well plate Gri3D® in combination with the MultiDrop and Viaflo (Figure 2). For liver spheroid aggregation, the optimal cell ratio is 10:3:1 (HB:ELC:HSC). Each well contains 73 micromolds and each sphere contains 3000 HB, 900 ELC and 300 HSC at the time of seeding. The cell density required for each cell type is described in the previous sections. The total volume needed per well is 40 μL but preparation of 60 μL per well is recommended. See Table 2 for details.

68. Calculate the number of wells needed and prepare accordingly to Table 2 in a 50 mL tube. As an example, to prepare a full 96-well plate, mix 3000 μL of HB with 1500 μL of ELC and 1500 μL of HSC.

***Note:*** Aggregation of liver spheres without ELC or HSC can be achieved by replacing the volume of the specific cell type within liver sphere medium supplemented with 10 μM Y-27632, 10 ng/mL EGF, 10 ng/mL FGF, 10 ng/mL HGF, 20 ng/mL OSM and 50 ng/mL VEGF. Liver function and long-term stability will be affected if ELC or HSC are removed.

69. Using the multidrop with a standard cassette, prime the cell solution until ready to dispense. Place a 96-well plate ‘V’-shape into the multidrop and dispense 60 μL at low speed.
70. Place the 96-well plate Gri3D® into position 1 of the Viaflo station (Figure 1B) in the plate holders of a three station Viaflo with a 96-well head. Place the plate with the A1 well facing the top left, the microwells (feeder wells) should be facing left at this position. Place a 96-deep well plate into the position 2 (Figure 1B) and use it as a waste and the ‘V’-shape plate with the cell suspension in the position 3 (Figure 1B).
71. Position the Viaflo head in the Gri3D® plate position, move the pin of the plate holder to the left to work in a 384-well plate mode. This mode should align the tips with the microwell of the 96-well plate Gri3D®.
72. Aspirate 300 μL to remove all the media from the feeder well. Move the Viaflo head into the central position where the waste deep-well plate is placed. Pin back to center to work in a 96-well plate mode and dispense the liquid into the waste plate. Remove any remaining liquid from the wells without damaging the hydrogel by touching it with the pipette tips and dispense the liquid into the waste plate.
73. Move the plate station to the right for the Viaflo to access the plate with the cells. Mix 4 times 50 μL at low speed (setting 3 in the Viaflo) and aspirate 40 μL. Move the plate station to the and place the Viaflo tips on top the hydrogel and dispense the 40 μL at speed 3. Move down to touch the dispensed liquid to fully dispense any remaining droplets in the tips. Dispense back any remaining cell suspension in the original plate with the cells.

***Note:*** If preparing multiple plates, dispense the total volume for all the plates a V-shape. This would reduce the extra volume needed.

74. Carefully remove the plate from the Viaflo and place it into the incubator at 37°C/5% CO_2_ for 2 h for cells to self-aggregate into spheres.
75. After 2 h, cells should be deposited within the micromold due to gravity. Place the 96-well plate Gri3D® into one of the plate holders of a three station Viaflo with a 96 well head. Using the multidrop, dispense 200 μL per well of liver sphere medium supplemented with factors as described before. If multiple plates have been prepared, dispense 200 μL per plate. Place the plate with the A1 well facing the top left, the microwells or feeder wells should be facing left at this position. Place a deep well 96-well plate in the next position, and the deep well with the media in the last position. Aspirate any liquid from the feeding chamber using the Viaflo and dispense it into the waste plate. Move the station for the Viaflo to access the plate with the media. Aspirate 150 μL, move back the plate station and move the Viaflo into the feeding chamber. Dispense the media at speed 3 and mix 100 μL 5 times at speed 5.

***Note:*** Incubation time can be extended up to 6 h to ensure cells are deposited into the micromolds. The previous step will be referred as media change for the 96-well plate Gri3D® plates

***Note:*** Automating the Viaflo pipetting of this process in custom programs is key to reduce variability. Automation optimization using a test plate and culture media without cells prior the sphere aggregation is recommended. Similar results can be achieved using a multichannel pipette but the use of semi-automatic liquid handling systems is recommended to reduce user-to-user and well-to-well variability.

**Figure 3:**
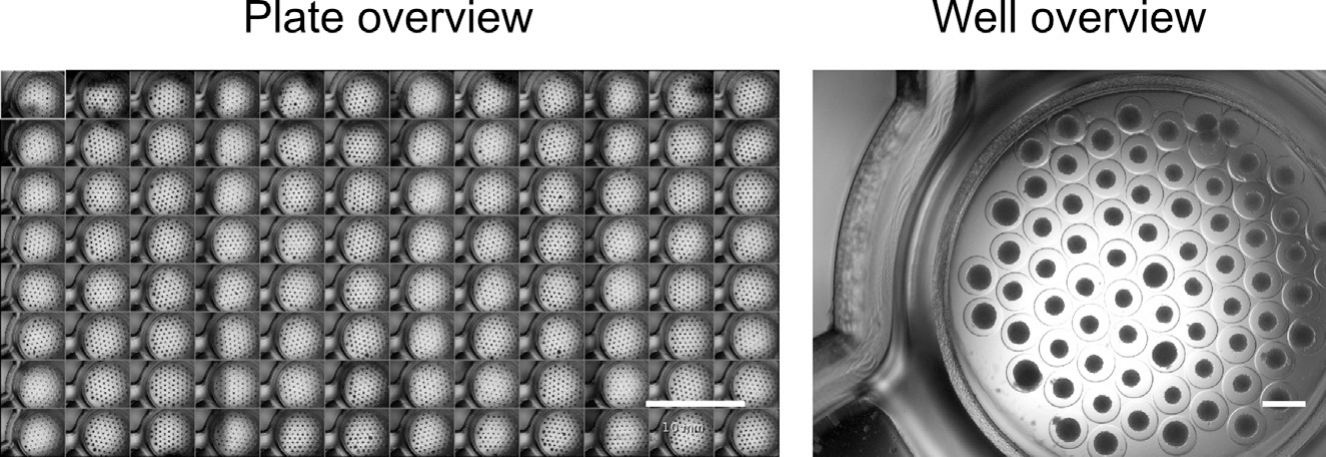
Overview of liver spheres in Gri3D® plate 96-well plate overview (left) with a scale bar of 10 mm; and well overview with liver spheres within the microwells (right) with a scale bar of 500 μm. Adapted from B Lucendo-Villarin et al., 2020 Biofabrication https://doi.org/10.1088/1758-5090/abbdb2.

### Measuring cytochrome P450 (CYP P450) metabolic activity in Gri3D® plates

**Timing: [1 h per medium change, 2 d per characterization]**

This step describes how to feed the liver spheres and characterize the cytochrome P450 for activity of liver spheres using the 96-well Gri3D® plate. Spheres can be maintained in this format up to one month.

76. Feed the plates every two to three days as described in the previous section. Aspirate 150 μL from the feeding chamber and replenish with liver sphere medium supplemented with 10 ng/mL EGF, 10 ng/mL FGF, 10 ng/mL HGF, 20 ng/mL OSM and 50 ng/mL VEGF.

***Note:*** Liver sphere medium in the wells can be collected prior to media change for downstream applications such as albumin or AFP secretion detection by enzyme-linked immunosorbent assay (ELISA) (Leedale et al., 2021, https://doi.org/10.1371/journal.pone.0244070).

***Note:*** Liver sphere medium supplemented with 10 ng/mL EGF, 10 ng/mL FGF, 10 ng/mL HGF, 20 ng/mL OSM and 50 ng/mL VEGF will be referred to as supplemented liver sphere medium.

77. Once a week, CYP P450 metabolic activity can be measured to assess liver sphere maturation. On a feeding day, prepare supplemented liver sphere medium with 100 μM of Luciferin-ME (Promega) to measure CYP P450 1A2 activity. Add the supplemented medium to the cells and incubate for 24 h.
78. Following incubation, use the Viaflo to mix 100 μL of the medium from the feeding chamber 5 times at speed 3 and aspirate 150 μL to mix completely the medium supplemented with Luciferin. Collect all medium from the feeding chamber using the Viaflo and dispense 50 μL per well into a 96-well white plates in triplicate. Feed the spheres with 150 μL per well with fresh liver sphere medium and place them back at the incubator.

**Pause point:** After dispensing of 50 μL into the white plates, plates can be frozen at −20°C and store for 1 month. The measurements can be performed at a later timepoint. For this, cover the plates with lids and seal with parafilm.

79. Add 50 μL per well of the luciferase reagent from the P450-Glo assay kit (Promega) into the white plates with the 50 μL of media with Luciferin-ME prepared previously and read the activity according to manufacturer's instructions.
80. Repeat the CYP P450 characterization once a week up to a month. On the last timepoint, after dispensing the media into the white plates, add 150 μL per well of RIPA buffer (Thermofisher) instead of supplemented liver spheroid media. Seal the plate with parafilm and freeze it at −20°C for at least 24 h.

***Note:*** At this step plates can be stored at −80°C for up to 3 months.

81. After 24 h, thaw the samples at room temperature (15°C–21°C) in a plate shaker at medium speed (∼ 30 rpm). Perform bicinchoninic acid assay (BCA, Thermofisher) protein quantification according to manufacturer's instructions. Use 10 μL of sample in triplicate for the BCA assay.

***Note:*** Plate sonication (23.3W) for 15 min can help to break down the structures of the spheres.

**Figure 4:**
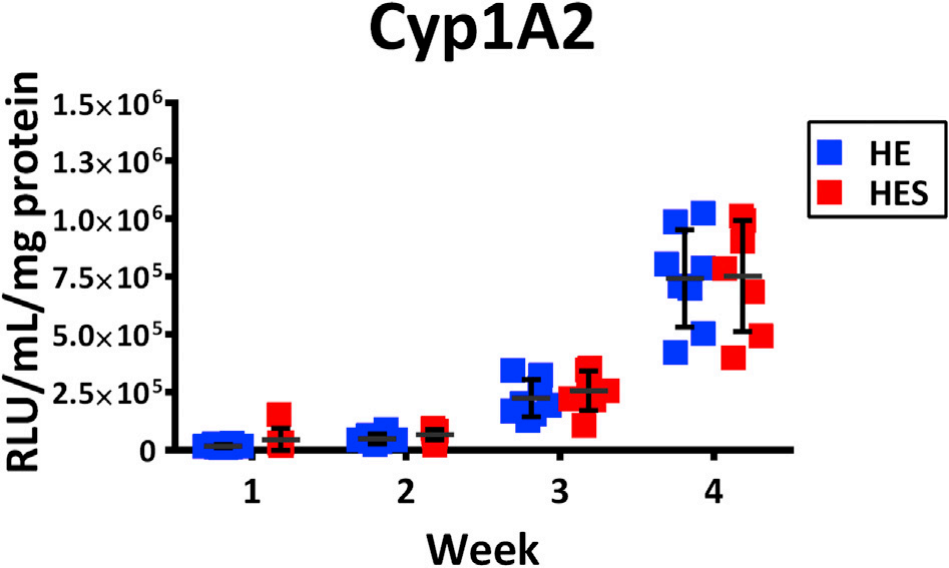
Cytochrome P450 1A2 activity in liver spheres at different time points (1, 2, 3 and 4 weeks) Abbreviations: HE – hepato-endothelial liver sphere, HES – hepato-endothelial-stellate liver sphere. Adapted from B Lucendo-Villarin et al., 2020 Biofabrication https://doi.org/10.1088/1758-5090/abbdb2.

82. Measure the relative levels of CYP P450 1A2 activity over time by normalizing the RLU signal per mg protein as determined by the BCA assay (Figure 4).

### Liver sphere morphological characterization by high content imaging in Gri3D® 96-well plates

**Timing: 2 h**

This step describes how to perform high content imaging of the liver spheres and the image analysis of the data generated. 48 h following liver sphere aggregation, morphological characterization of liver sphere size and number of spheres per well can be performed using the Operetta high content imaging system (PerkinElmer). This procedure offers a non-disruptive method for sphere size characterization which can be used to monitor sphere growth under normal cell culture conditions or during screening experiments.

**Figure 5:**
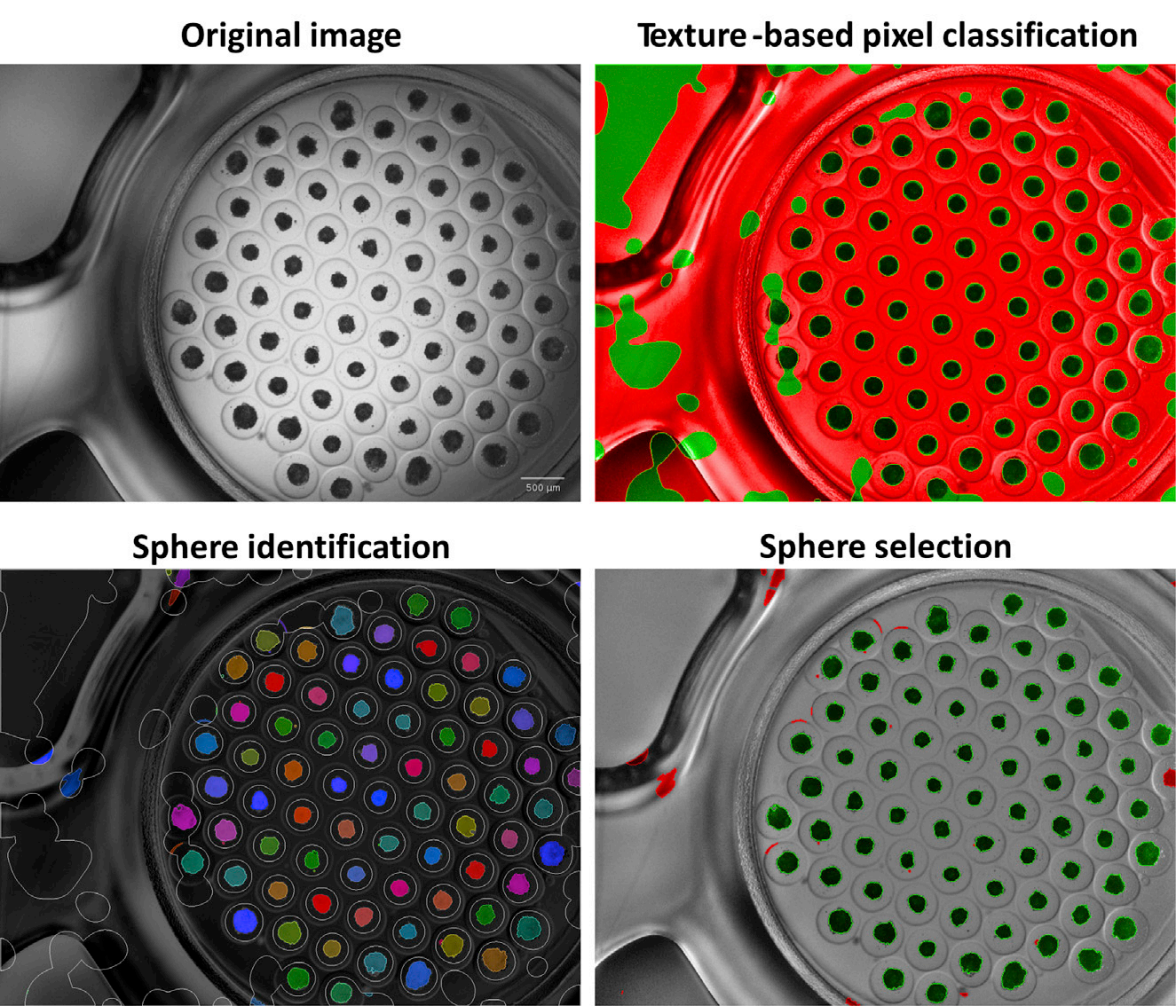
Label-free sphere segmentation The 96-well plates were imaged using the Operetta high content imaging system. Most of the well with the liver spheres was captured using 2**×** objective. Image was analyzed using Columbus software. Texture-based pixel classification was performed for sphere identification and background removal. An inverted image was used for sphere identification based on sphere size and texture using ‘Find Nuclei’ function. For quality control step, an object classification was employed to select only properly segmented spheres. Adapted from B Lucendo-Villarin et al., 2020 Biofabrication https://doi.org/10.1088/1758-5090/abbdb2.

**Figure 6:**
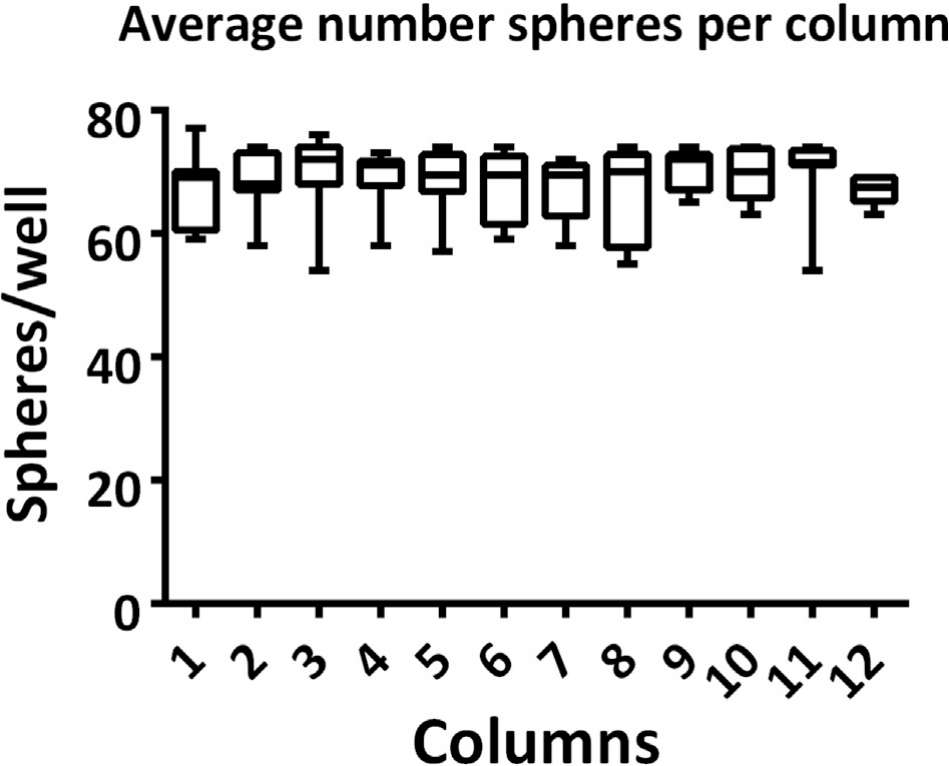
Number of spheres per column of 96-well plate (mean ± SD, n = 8) Spheres were counted automatically using the image analysis pipeline described in Data S1**: Material analysis pipeline - Analysis Sequence ‘SphereQuant_texture’**, related to step 85. Adapted from B Lucendo-Villarin et al., 2020 Biofabrication https://doi.org/10.1088/1758-5090/abbdb2.

***Note:*** Similar results can be achieved with open-access software such as Ilastik or CellProfiler (Carpenter et al., 2006, https://doi.org/10.1186/gb-2006-7-10-r100; Sommer et al., 2011, https://doi.org/10.1109/ISBI.2011.5872394).

83. If your high content imaging system is equipped with a live chamber to maintain the cells at 37°C/5% CO_2_, precondition the live imaging chamber 30 min before imaging. Place the plate on the live imaging chamber once it reached the right temperature and CO_2_ concentration.
84. Using a 2**×** objective, most of the well containing the liver spheres should be captured in a single image. Set the focus and image the whole plate. After imaging, place the plate back to the incubator.
85. Following image acquisition, image analysis can be performed using Columbus software (Data S1**: Material analysis pipeline - Analysis Sequence ‘SphereQuant_texture’**). First, use texture-based pixel classification to identify the liver spheres from the background. This can be achieved by training texture-based classifier to distinguish between two classes (cell aggregate and background) by selecting multiple examples of pixels for each class (Figure 5). Next, generate an inverted image which can be used for sphere identification based on sphere size and texture using the ‘Find Nuclei’ block (Figure 5). Following this, an object classification can be used for sphere selection as a quality control step to ensure that only properly segmented spheres are analyzed (Figure 5). Finally, object count, and morphology quantification are performed for sphere count and area quantification (Figure 6).

## Expected outcomes

This protocol generates stem cell derived liver spheres in a 96-well format using a semi-automated pipeline. By combining stem cell derived hepatic progenitors, endothelial cells, and hepatic stellate cells, a model system that better recapitulates liver biology can be generated. Figure 3 shows a plate and well overview of the liver spheres following sphere aggregation, and high content imaging can be used as a non-invasive method of sphere characterization (Lucendo-Villarin et al., 2020). Figure 5 shows an overview of the high content analysis pipeline for sphere segmentation. See Data S1: Material analysis pipeline - Analysis Sequence ‘SphereQuant_texture’, related to step 85, for further details for the image analysis. Following image analysis, quantification of the sphere number per well can be used to assess variability (Figure 6). The liver spheres produced by this protocol display increasing metabolic activity (Cyp 1A2) from one week following sphere aggregation up to a month (Figure 4). These spheres are functional and phenotypically stable when manufactured at scale, creating a powerful tool for disease modeling and drug screening (Leedale et al., 2021; Lucendo-Villarin et al., 2020).

## Limitations

At this stage, the three cell types nor their corresponding liver spheres, can be cryopreserved. If using different cell lines, optimization of cell seeding, prior to differentiation, might be required. To optimize the cell density try adding or removing 10,000 cells/cm2 at a time.

## Troubleshooting

### Problem 1

Step 24 - Following single cell seeding, cell distribution is not homogeneous, leading to a heterogenous differentiation.

### Potential solution

Cell attachment to LN-521 coated plates occurs promptly following cell seeding. To ensure that the single cell suspension is dispersed evenly across the well or petri dish gently agitate the plate back-and-forth and from side-to-side multiple times (>10**×**).

### Problem 2

Step 46 - Single cell detachment is not achieved following the TrypLE incubation time.

### Potential solution

Before adding the TrypLE, ensure cells are properly washed with 1**×** DPBS as this can be cell line dependent. Increase the incubation time for an additional 5 min.

### Problem 3

Steps 35, 47, and 58 - Filtering the single cell suspension in a 30 μm filter takes too long (>5 min) or filters get clogged.

### Potential solution

Pre-filter the cell suspension using a 100 μm filter or change the 30 μm filter.

### Problem 4

Steps 63**–**65 - MACS endothelial enrichment takes too long (>20 min).

### Potential solution

Use multiple MACS columns to reduce purification times or use MACS cell separation instruments with greater capacity

### Problem 5

Step 81 - The cell suspension leaks from the seeding well into the feeding chamber following dispensing into the 96-well Gri3D.

### Potential solution

Ensure both feeding chamber and seeding well are completely dry prior to dispensing cells. Following cell seeding, remove the plate from the Viaflo plate holder and carefully place it into the incubator. Any sudden movements of the 96-well plate Gri3D could lead to cell suspension leakage from the seeding chamber.

## Resource availability

### Lead contact

Further information and requests for resources and reagents should be directed to and will be fulfilled by the lead contact, David C. Hay (davehay@talktalk.net).

### Materials availability

This study did not generate new unique reagents. Original materials can be found at B Lucendo-Villarin et al., 2020 Biofabrication https://doi.org/10.1088/1758-5090/abbdb2.

### Data and code availability

This study did not generate/analyze any new data. Original data can be found at B Lucendo-Villarin et al., 2020 Biofabrication https://doi.org/10.1088/1758-5090/abbdb2.

## Supplemental information

Data S1. Material analysis pipeline, related to step 85
