# Supplementary material for: Protocol for automated production of human stem cell derived liver spheres
Source: STAR Protoc. 2021 Apr 30;2(2):100502. doi: 10.1016/j.xpro.2021.100502 (PMC8105683; doi:10.1016/j.xpro.2021.100502)
Supplement: Data S1. Material analysis pipeline, related to step 85 [file mmc1.pdf]

Analysis Sequence "SphereQuant_texture"

Input Image	Input
	**Flatfield Correction :** None<br>Brightfield Correction<br>**Stack Processing :** Individual Planes<br>**Min. Global Binning :** Dynamic

Filter Image	Input	Method	Output
	**Channel :** Brightfield	**Method :** Invert Image<br>Cut-off Quantile : 100	Output Image : Inverted

Find Texture Regions	Input	Method	Output
	**Channel :**<br>**ROI :** None	**Method :** Split into Classes<br>Number of Classes : 2<br>Texture Scale : <u>20</u> px<br>Region Scale : <u>20</u> px<br>Training Region Radius : <u>10</u> px	Output Population A : Pre-sphere<br>Output Population B : Texture B

Modify Population	Input	Method	Output
	**Population :** Pre-sphere<br>**Region :** Region	**Method :** Cluster by Distance<br>Distance : 0 px<br>Area : > 0 px²<br>Fill Holes	Output Population : Modified Pre-sphere<br>Output Region : Region

Select Region	Input	Method	Output
	**Population :** Pre-sphere<br>**Region :** Region	**Method :** Resize Region [%]<br>Outer Border : -30 %<br>Outer Population : None<br>Outer Region :<br>Inner Border : 100 %	Output Region : Region Resized

Find Nuclei	Input	Method	Output
	**Channel :** Inverted<br>**ROI :** Pre-sphere<br>**ROI Region :** Region Resized	**Method :** B<br>Common Threshold : <u>0.2</u><br>Area : > <u>300</u> µm²<br>Splitting Coefficient : <u>9.5</u><br>Individual Threshold : <u>0.75</u><br>Contrast : > <u>0.05</u>	Output Population : Sphere

Modify Population (2)	Input	Method	Output
	**Population :** Sphere	**Method :** Cluster by	Output Population :

	**Region :** Nucleus	Distance<br>Distance : 0 px<br>Area : > 0 px²<br>Fill Holes	Modified Sphere<br>Output Region : Region

**Select Region (2)**	**Input**	**Method**	**Output**
	**Population :** Modified Sphere<br>**Region :** Region	**Method :** Resize Region [%]<br>Outer Border : -10 %<br>Outer Population : None<br>Outer Region :<br>Inner Border : 100 %	Output Region : BigSphere

**Calculate Intensity Properties**	**Input**	**Method**	**Output**
	**Channel :** Brightfield<br>**Population :** Modified Sphere<br>**Region :** BigSphere	**Method :** Standard<br>Mean<br>Standard Deviation<br>Coefficient of Variance<br>Median<br>Sum<br>Maximum<br>Minimum<br>Quantile Fraction : 75 %<br>Contrast	Property Prefix : Intensity BigSphere Brightfield

**Calculate Position Properties**	**Input**	**Method**	**Output**
	**Population :** Modified Sphere<br>**Region :** BigSphere	**Method :** Standard<br>Centroid Position in Image<br>Nearest Neighbor Distance<br>Contact Area with Image Border<br>Contact Area with Neighbors	Property Prefix : BigSphere

**Calculate Morphology Properties**	**Input**	**Method**	**Output**
	**Population :** Modified Sphere<br>**Region :** BigSphere	**Method :** Standard<br>Area<br>Roundness<br>Width<br>Length<br>Ratio Width to Length	Property Prefix : BigSphere

**Calculate Texture Properties**	**Input**	**Method**	**Output**
	**Channel :** Brightfield<br>**Population :** Modified Sphere	**Method :** SER Features<br>Scale : 0 px<br>Normalization by : Kernel	Property Prefix : BigSphere Brightfield

	**Region :** BigSphere	SER Spot<br>SER Hole<br>SER Edge<br>SER Ridge<br>SER Valley<br>SER Saddle<br>SER Bright<br>SER Dark	

**Select Population**	**Input**	**Method**	**Output**
	**Population :** Modified Sphere	**Method :** Linear Classifier<br>Number of Classes : 2<br>Intensity BigSphere Brightfield Mean<br>Intensity BigSphere Brightfield StdDev<br>Intensity BigSphere Brightfield Median<br>Intensity BigSphere Brightfield Maximum<br>Intensity BigSphere Brightfield Minimum<br>Intensity BigSphere Brightfield Sum<br>Intensity BigSphere Brightfield CV [%]<br>Intensity BigSphere Brightfield Quantile 75%<br>Intensity BigSphere Brightfield Contrast<br>BigSphere Centroid X in Image [µm]<br>BigSphere Centroid Y in Image [µm]<br>BigSphere Nearest Neighbor Distance [µm]<br>BigSphere Contact Area with Image Sides [%]<br>BigSphere Contact Area with Neighbors [%]<br>BigSphere Area [µm²]<br>BigSphere Roundness<br>BigSphere Width [µm]<br>BigSphere Length [µm]<br>BigSphere Ratio Width to Length<br>BigSphere Brightfield SER Spot 0 px<br>BigSphere Brightfield SER Hole 0 px<br>BigSphere Brightfield SER Edge 0 px<br>BigSphere Brightfield SER Ridge 0 px<br>BigSphere Brightfield SER Valley 0 px<br>BigSphere Brightfield SER Saddle 0 px<br>BigSphere Brightfield SER Bright 0 px<br>BigSphere Brightfield SER Dark 0 px	Output Population A : Sphere Selected<br>Output Population B : Class B

Define Results	Results
	**Method :** List of Outputs<br>**Population : Texture B**<br>Number of Objects<br><br>**Population : Sphere Selected**<br>Number of Objects<br>BigSphere Area [μm²] : Mean+StdDev<br><br>**Object Results**<br>Population : Texture B : None<br>Population : Modified Pre-sphere : None<br>Population : Pre-sphere : None<br>Population : Modified Sphere : None<br>Population : Sphere : None<br>Population : Sphere Selected : None<br>Population : Class B : None

Acapella version: 5.1.1.126729. Timestamp: 2020-12-04 16:58:26 +0000.
